# Tissue Engineered Scaffolds in Regenerative Medicine

**Published:** 2014-01

**Authors:** Mohsen Hosseinkhani, Davood Mehrabani, Mohammad Hassan Karimfar, Salar Bakhtiyari, Amir Manafi, Reza Shirazi

**Affiliations:** 1 Department of Anatomical Sciences, School of Medicine, Qazvin University of Medical Sciences, Qazvin, Iran;; 2 Stem Cell and Transgenic Technology Research Center, Department of Pathology, Shiraz University of Medical Sciences, Shiraz, Iran;; 3 Department of Anatomical Sciences, School of Medicine, Zahedan University of Medical sciences, Zahedan, Iran;; 4 Department of Clinical Biochemistry, School of Medicine, Ilam University of Medical sciences, Ilam, Iran;; 5 Student Research Committee, Shahid Beheshti University of Medical Sciences, Tehran, Iran;; 6 Nanobiotechnology Research Center, Avicenna Research Institute, ACECR, Tehran, Iran

**Keywords:** Stem cells, Tissue engineering, Scaffold, Regenerative medicine

## Abstract

Stem cells are self-renewing cells that can be differentiated into other cell types. Conventional *in vitro* models for studying stem cells differentiation are usually preformed in two-dimensional (2D) cultures. The design of three-dimensional (3D) *in vitro* models which ideally are supposed to mimic the* in vivo* stem cells microenvironment is potentially useful for inducing stem cell derived tissue formation. Biodegradable scaffolds play an important role in creating a 3D environment to induce tissue formation. The application of scaffolding materials together with stem cell technologies are believed to hold enormous potential for tissue regeneration. In this review, we provide an overview of application of tissue engineered scaffolds and stem cells for the development of stem cell-based engineered tissue replacements. In particular, we focus on bone marrow stem cells (BMSCs) and mesenchymal stem cell (MSCs) due to their extensive clinical applications.

## INTRODUCTION

Stem cells are primitive cells found in many multi-cellular organisms and possess self-renewal and potency abilities. Self-renewal is that characteristic of stem cells that maintains them in numerous cell cycle divisions, while potency defines the differentiation capability of stem cells into mature cell types. Mammalian stem cells are categorized based on the source they are derived from: embryonic stem (ES) cells, derived from blastocysts, and adult stem cells, found in adult tissues.^1^^-^^5^

Embryonic Stem cells (ES) have the potential to differentiate into a number of different cell types. ES cell differentiation can be induced from cell aggregates, called embryonic bodies (EBs), which initiate many developmental processes and generate derivatives of the three primary germ layers (i.e. ectoderm, mesoderm, and endoderm).^2^^,^^6^^-^^8^ Because of their ability to differentiate into all the cell types of an adult organism, ES cells are useful for cell-replacement therapies for a number of diseases including Alzheimer’s disease, Parkinson’s disease, spinal cord injury, diabetes and infarcted heart.^9^^-^^12^

Adult stem cells are a class of stem cells comprised of undifferentiated cells found in many tissues of an adult organism and have an extensive self-renewal capability and the ability to differentiate into various specialized cell types (i.e. blood, muscle, and nerve cells).^13^^-^^17^ Bone marrow contains at least two kinds of stem cells. One population, called hematopoietic stem cells, form all types of blood cells in the body. A second population, called bone marrow stromal cells, was discovered a few years later. Bone marrow stromal cells (mesenchymal stem cells) give rise to a variety of cell types: bone cells (osteocytes), cartilage cells (chondrocytes), fat cells (adipocytes), and other kinds of connective tissue cells such as those in tendons.^2^^,^^3^ The primary roles of adult stem cells in a living organism are to maintain and repair the tissue in which they are found and exhibit the ability to form specialized cell types of other tissues, which is known as transdifferentiation or plasticity**.**^13^^,^^18^

Because functional tissues require certain mechanical and structural properties; tissue engineering has aimed at performing specific biochemical functions using cells in artificial support systems. One should consider the fact that generating cell-laden tissue engineered constructs has the capability of maintaining the proliferation and differentiation activity. Therefore, design of engineered tissues from cell proliferation and differentiation is one of the key objectives of tissue engineering.^19^ Considering the usage of cells in the body, it is no doubt that a sufficient supply of nutrients and oxygen to the transplanted cells is vital for their survival and functional maintenance.^20^^,^^21^ Also without a sufficient supply, only a small number of cells pre-seeded in the scaffold or migrated into the scaffold from the surrounding tissue would survive. In conventional cell culture such as static tissue culture dish (2D), the initial rate of cell growth is higher, but the proliferation stops once the cells reach confluence. In order to overcome supply limitations, porous materials with (3D) structures have been generated because of their larger surface for cell attachment and proliferation relative to 2D materials. They are also preferable in assisting with the formation of 3D cell constructs which may resemble the structure and function of body tissues. Moreover, diffusion of nutrients, oxygen and bioactive factors through 3D constructs is sufficient for survival of large numbers of cells for extended periods of time.^22^^,^^23^

Also presence of extracellular matrix (ECM) and soluble factors (that induce vascuarization and differentiation of stem cells embedded into these constructs) addresses some of the limitations for application of tissue engineering constructs. Rapid formation of a vascular network at the transplanted site of cells seems promising for providing stem cells with the vital supplies. This process of generating new microvasculature, termed neovascularization, is a process observed physiologically in development and wound healing.^22^ Previous reports indicated basic fibroblast growth factor (bFGF) promoting angiogenesis process.^22^^,^^24^ The growth factors stimulate the appropriate cells (e.g., endothelial cells), already present in the body, to migrate from the surrounding tissue, proliferate, and finally differentiate into blood vessels. However, bFGF has a very short half-life when injected and is unstable in solution. To overcome these problems, bFGF was encapsulated within alginate, gelatin, agarose/heparin, collagen, and poly (etyhylene-co-vinyl acetate) carriers.^25^^,^^26^ One possible way for enhancing the* in vivo* efficacy is to achieve its controlled release over an extended time period by incorporating the growth factor in a polymer carrier. If this carrier is biodegraded, harmonized with tissue growth, it will work as a scaffold for tissue regeneration in addition to a carrier matrix for the growth factor release. 

Synthetic materials demonstrate interesting characteristics since their chemical and physical properties (e.g., porosity, mechanical strength) can be specifically optimized for a particular application. The Structure of polymeric scaffolds are endowed with a complex internal architecture and porosity that provide sites for cell attachment and maintenance of differentiation function without hindering proliferation.^27^ Ideally, a polymeric scaffold in tissue engineering should have the following characteristics: i) Holding appropriate surface properties promoting cell adhesion, proliferation and differentiation; ii) Biocompatiblity; iii) High porosity, and a high surface-area to volume ratio, with an interconnected pore network for cell growth and flow transport of nutrients and metabolic waste, iv) Sufficient mechanical properties and any* in vivo* stresses.^27^^-^^31^ Xie *et al*. reported that the initial rate of cell growth is higher for the 2D culture, but once the cells reach confluence, their proliferation stops.^32^ Other reports have also demonstrated that cell proliferation in the 3D scaffolds is superior to the 2D scaffolds. Therefore, the cell growth in the 3D scaffolds continues for longer time periods than that of 2D scaffolds.^33^^,^^34^

The design of materials that can regulate cell behavior such as proliferation and differentiation is a key component for the fabrication of tissue engineering scaffolds. From the view point of immune system response of the body, the implanted biomaterials should mimic the structure and biological functionality of native extracellular matrix (ECM), both in terms of chemical composition and physical structure as reported by Ma *et al*.^35^ Therefore, the scaffold materials used in tissue engineering need to be chemically functional in order to promote tissue regeneration as ECM does. ECM proteins such as Collagen and Elastin are made from fibers in dimensions smaller than micrometers.^35^ It seems that artificial nanoscale fibers have great potential application in the field of biomaterials and tissue engineering. The initial report showed that nanoscale features influence cell behaviors.^36^ Nanoscale surface topography has been found to promote osteoblast adhesions.^37^ It has been demonstrated that osteoblast adhesion, proliferation, alkaline phosphatase activity, and ECM secretion on carbon nanofibers increases with decreasing fiber diameter in the range of 60-200 nm, whereas the adhesion of other kinds of cells such as chondorocytes, fibroblasts, and smooth muscle cells remains non-influenced.^38^^,^^39^ It has been believed that the nanoscale surfaces affect the conformation of adsorbed adhesion proteins such as vitronectin, thus affecting the cell behaviors. In addition, the nanoscale dimensions of cell membrane receptors such as integrins should also be considered.^40^

Cell proliferation in 3-D scaffold, needs oxygen and nutrition supply. Therefore, the 3-D scaffold materials should provide such an environment for cells. The artificial scaffolds formed by self-assembling molecules not only provide suitable support for cell proliferation but also serve as a medium through which diffusion of soluble factors and migration of cells can occur. The result of the cell attachment and proliferation revealed that diffusion of nutrients, bioactive factors, and oxygen through these highly hydrated networks is sufficient for survival of large numbers of cells for extended periods of time.^26^

The 3D scaffolds are capable of differentiating a single progenitor cell population into particular lineage either due to bulk incorporation of soluble factors within the scaffolds or due to exogenous delivery of chemicals, hormones, and growth factors in culture medium. Therefore, design of patterned scaffolds with the ability to develop multiple lineages and hybrid organ structures could provide promising alternatives.^26^

Metal scaffolds like titanium are bio-compatible and suitable for hard-tissue applications, such as the growth and differentiation of rat dental pulp progenitor cells into odontoblast-like cells. In order to improve their efficacy, metal scaffolds can be covered with biological compounds, like titanium fibers pre-coated with ECM components that support the osteogenic differentiation of rats’ BMSCs.^41^^-^^43^

Another type of scaffolds is made of organic materials that provide a bio-mimetic environment for stem cells. Human BMSCs regenerate bone in marine sponge skeletons, cartilage in silk fibroin scaffolds, and adipose tissue in gelatin. To provide mechanical strength, biological agents influencing stem cell fate could be added to the scaffold’s compounds. Marine sponge skeletons, for example, contain these cell adhesion proteins: fibronectin, collagen and gelatin.^44^^,^^45^

Synthetic polymers provide greater control over the mechanical and degradation properties of the tissue scaffold. For example, self-assembling peptide scaffolds contain highly flexible nano-fibers that support the differentiation of rat liver progenitor cells into functional hepatocyte-like spheroinods.^26^ It has been reported that the attachment, proliferation and ECM production of human and rat BMSC, are promoted while copolymers of poly (e-caprolactone) and poly (ethylene glycol) are blocked. Ligand binding and controlled release of regulatory factors can improve the degree of control over spatial organization and differentiation of stem cells within scaffolds. Osteogenic differentiation of rat BMSCs was enhanced into an oligo (polyethylene glycol) scaffold that incorporated peptides containing the cell-binding sequence arginine-glycine-aspartate in the absence of soluble factors like dexamethasone. Moreover, controlling the release of regulatory molecules from porous poly (D,L-lactideco- glycolide) scaffold inducing rabbit BMSCs within the scaffold to mineralize* in vitro *into bone.^42^^,^^44^

The application of scaffolding materials together with stem cell technologies are believed to hold enormous potential for tissue regeneration. In this review, we provide an overview of application of tissue engineered scaffolds and stem cells for the development of stem cell-based engineered tissue replacements. In particular, we focus on bone marrow stem cells (BMSCs) and mesenchymal stem cell (MSCs) due to their extensive clinical applications. 

## Footenote

Please cite this paper as:

Hosseinkhani M, Mehrabani D, Karimfar MH, Bakhtiyari S, Manafi A, Shirazi R. Tissue Engineered Scaffolds in Regenerative Medicine. World J Plast Surg 2014;3(1):3-7.
